# Dental service for United Nations peacekeepers coping with COVID-19 in Bukavu: preparation, implementation and recommendation

**DOI:** 10.3389/froh.2025.1527777

**Published:** 2025-02-20

**Authors:** Shuai Lu, Li Miao, Yong He, Jia-Ning Luo, Lu Lin, Zhi-Hua Liu, Bi-Yu Yan, Jia-Ling Wu, Yang Xie, Shu-Yong Yang, Chao Wang

**Affiliations:** ^1^ Department of Stomatology, General Hospital of Western Theater Command PLA, Chengdu, China; ^2^ Chinese Level 2 Hospital, Bukavu, Democratic Republic of Congo; ^3^ Department of Stomatology, The Seventh Medical Center of PLA General Hospital, Beijing, China; ^4^ Department of Neurosurgery, General Hospital of Western Theater Command PLA, Chengdu, China; ^5^ Department of Anesthesiology, General Hospital of Western Theater Command PLA, Chengdu, China; ^6^ Department of Stomatology, The 955th Hospital of PLA, Changdu, China; ^7^ Ministry of Health, Xining Joint Logistics Support Center, Xining, China; ^8^ Department of Radiology, General Hospital of Western Theater Command PLA, Chengdu, China; ^9^ Health Service Training Center, General Hospital of Western Theater Command PLA, Chengdu, China

**Keywords:** peacekeeping, MONUSCO, COVID-19, dental service, treatment needs

## Abstract

**Background:**

The Chinese level 2 hospital (CHH L2) deployed in Bukavu provides medical supports to peacekeepers in MONUSCO. This study aimed to statistically analyze the types of oral and maxillofacial problems and corresponding treatments provided from October 2018 to September 2022, and to describe the trends of dental service pre and post COVID-19 outbreak.

**Methods:**

The medical records of all patients visited to the CHN L2 during the 48 months were collected and were accessed for the research purposes between October 2018 and September 2022. Dental visitors were counted and identified with nationality and occupations. Dental service was categorized as emergency, routine and evacuation. Diagnosis and treatments provided were statistically analyzed.

**Results:**

952/3,913 (24.33%) of the visitors to CHN L2 during this period were referred to dentistry, including 50 females (5.25%) and 902 males (94.75%). The proportion of UN military personnel is 91.39% (870/952). A total of 1,116 teeth and mucosa problems were treated. Dental emergencies represented 13.98% percent (156/1, 116). 2,180 dental treatments (2.29 procedures per patient) were provided: dental radiographs taken (618, 28.30%), local anesthesia (448, 20.55%), RCT (373, 15.14%), resin composite filling (330, 15.14%), extraction (248, 11.38%) and other treatments (164, 7.52%). The number of monthly visitors was significantly affected by the COVID-19 epidemic (*p* < 0.05), pre-COVID-19 (total 151 ± 51, dental 33 ± 13) and post-COVID-19 (total 51 ± 21, dental 13 ± 8), the lowest number was in July 2020, 6 months after the COVID-19 outbreak (total 16 and dental 3).

**Conclusions:**

Dentistry is the most frequently visited department in the CHH L2 in Bukavu, and the number of visitors were affected by the COVID-19 epidemic. Treatments related to dental caries, pulpitis, apical periodontitis, and wisdom teeth problems account for the majority of dental service. The variety and severity of dental problems demonstrate the urgent need for training in multiple specialized skills for deployed military dental personnel.

## Background

The Democratic Republic of Congo (DRC) has experienced decades of armed conflicts, insecurity and forced displacement, particularly in its eastern regions (1). With an extended mandate to support stabilization and consolidation of peace and to protect civilians in the DRC, MONUSCO, one of the most extensive peace support operations in history, currently consisting of almost 17,700 actively deployed personnel, took over from the United Nations Organization Mission in DRC (MONUC) on 1st July 2010 (2). Despite some improvements in the security situation, armed conflicts, violence, poverty and diseases remained endemic and pervasive, the health status of the peacekeepers in South Kivu remains extensive epidemiological threatened, including of all cases of major infections, like Ebola, malaria and the increased coronavirus disease 2019 (COVID-19) (3–5). The outbreak of COVID-19 has resulted in enormous loss of human lives and economy worldwide, and continues to severely impact people's health, as well as military operations. This highly contagious characteristics of the coronavirus through respiratory transmission and aerosol especially produced during dental treatments increased occupational risk for medical staffs and the risk of infection to patients in waiting areas and their comrades in camp (6, 7).

As one of the subordinate medical units of MONUSCO, the Chinese level II hospital (CHH L2) in Bukavu, South Kivu, undertakes the mission of medical service to UN civilian staffs and military personnel deployed in their local areas of responsibility. Dental service in CHH L2 was categorized as emergency, routine, hospitalization or evacuation due to maxillofacial trauma or other reasons. It is also necessary to formulate appropriate and flexible strategies of admission and discharge of COVID-19 patients at CHH L2 to cater to the healthcare needs. Studies have reported advise of dental service procedures and managements in public dental service and clinics before or post COVID-19, and gave advises in an emergency or routine condition (8–10). We learned from experiences about the prevention and control of respiratory borne diseases and transformed the dental office in the CHH L2 to satisfy the special peacekeeping mission, and that was successfully passed the Continent-Owned Equipment Inspection (COEI) and Operational Effectiveness Inspection (OEI) of MONUSCO.

Unlike other medical facilities in the world, the UN Hospitals established for peacekeeping missions are characterized by annual rotation of medical staff, shortage of manpower, limited supply of medical supplies, and backward infrastructure (11). It is very important to estimate how many troops and/or staffs may need dental care, and which months are the peak times and what kinds of dental treatments might be required during the peacekeeping missions. All of this information is useful for the dentist to organize the upcoming dental service. Having an accurate understanding of the types of oral and maxillofacial diseases and corresponding treatment workload during the mission is very useful for medical team leaders to formulate division of labor, the number of dental assistants, and the carrying of medical equipment and consumables (12). This study described the trends and experience of CHH L2 dental care service for MONUSCO before and after the COVID-19 outbreak from October 2018 to September 2022, classified the diseases of dental patients and related treatment items, and made recommendations for subsequent dentists and medical team leaders.

## Methods

All patients involved in this retrospective study provided written consent for this anonymized research. The examinations complied with the requirements set by the Ethics Committee of the Seventh Medical Center of PLA General Hospital. And the Number of ethics committee approval is No. 2018-076.

### Set-up of the dental care room at CHH L2

The CHH L2 medical team members rotate in every September, one dentist is assigned for each mission period. According to guidelines of medical support for UN peacekeeping operations (13, 14), dental unit should have the capacity to perform 10 basic dental services every day. As a field hospital built by movable plank houses, there was only one dental chair comes with a deionized water tank in an independent treatment room was set in CHH L2. The air compressor and a store cabinet for all the dental materials were arranged in the same room. One x-ray machine was enclosed by a lead plate, and the digital acquisition system (RVG5200) is used. The x-rays were displayed on computer screen for dentist to view and communicate with patients. The technical laboratory and sterilizing room were shared with other departments. Another x-ray machine and two air compressors backup were reserved for backup in the warehouse.

**Figure 1:**
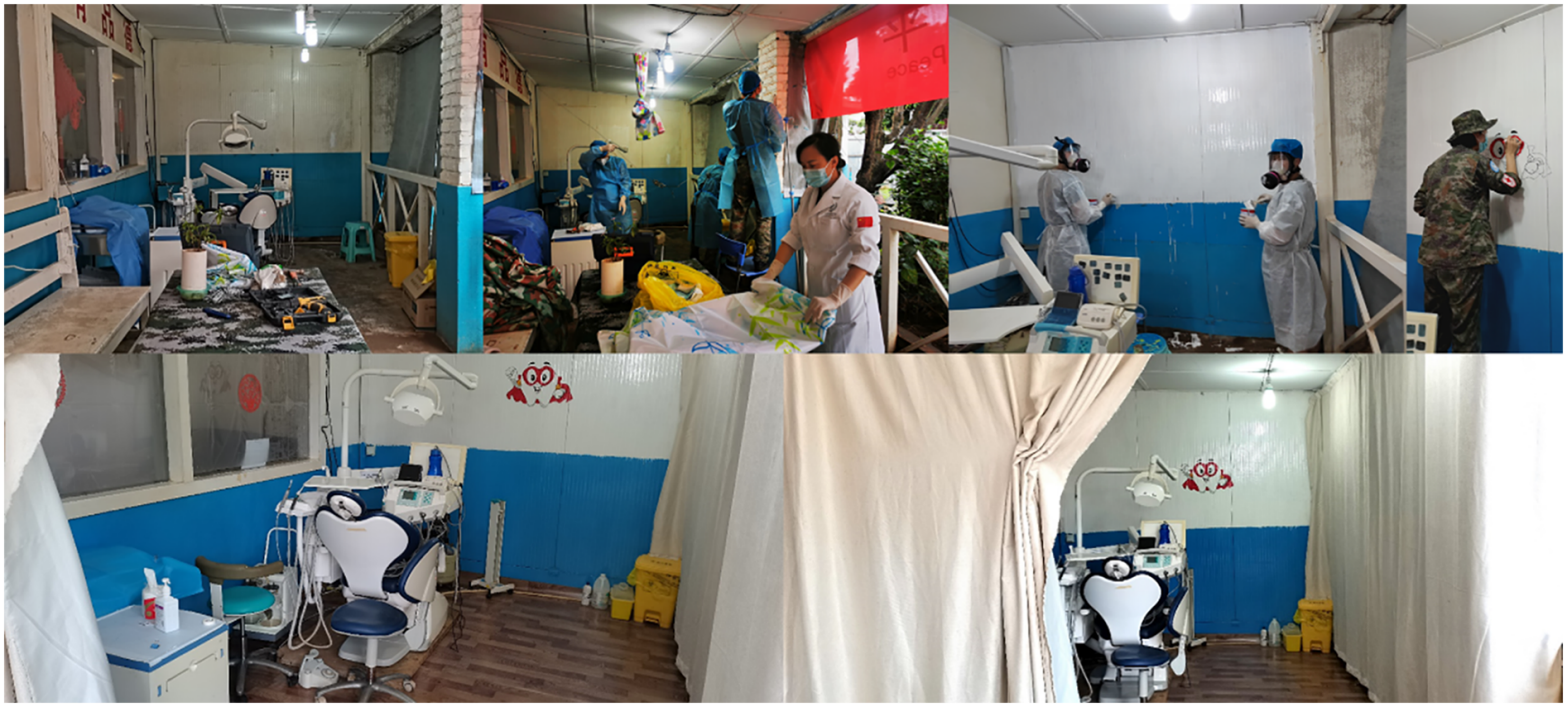
Renovation of dental clinic. Original condition. Refurbishment, brush the wall, painting, decoration completed. Outside view.

After the COVID-19 outbreak in Jan 2020, dental room was relocated from the interior of the facility to a corner of the outdoor corridor, considering the generation of high amounts of droplets and aerosols during routine dental procedures which was proved to be effective in spreading of COVID-19 (15, 16). After laying the ground, painting the walls, setting up enclosures and adding rain cloths, a simple dental care room configurated with dental chair, air compressor unit, negative pressure suction device unit, operating table, material cabinet, medical trash can, Ultraviolet disinfection machine and Uninterruptible Power Supply (UPS) was completed, ensuring the area meets privacy, facilitates ventilation and sterilization (Figure 1).

### Visiting procedures

**Figure 2:**
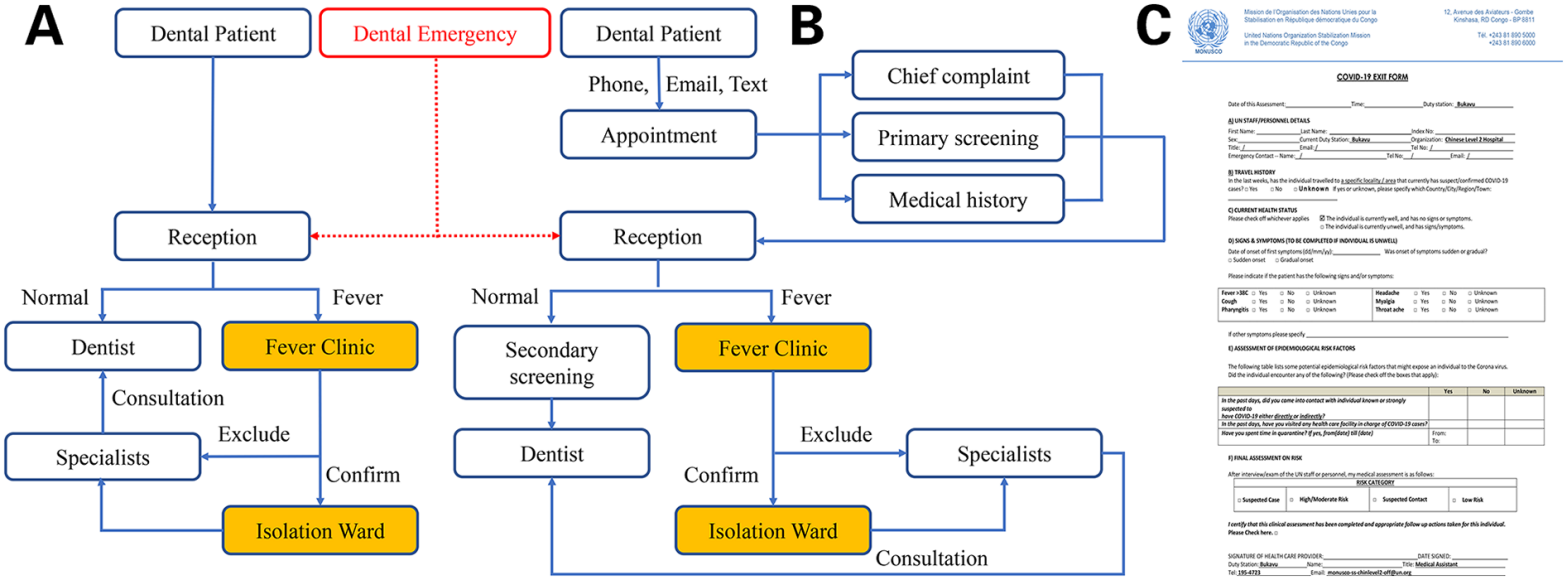
Dental patient visit process. **(A)** Pre-COVID-19; **(B)** post-COVID-19; **(C)** COVID-19 questionnaire.

Every peacekeeper or staff working in MONUSCO has a unique ID certificate. A referral form from the Level-1 hospital and the ID card were required when he or she comes to CHH L2 for treatment. Every patient seeking for medical care in CHH L2 was first registered at the reception desk. The receptionist will fill out a health form, including ID number, name, sex, unit, rank, nationality, and health status, and then copy the ID card and create a unique serial number for each patient. Multiple visits of the same patient were recorded separately and add new serial number. The patient was then escorted into the treatment room by an assistant. After the dental examination or treatment, the dentist filled the brief findings in the medical record. The detail information of treatments was recorded in an invoice form including description, fee, number, date, and patient's signature, and then sign the name of dentist. The patient file contains referral form, health form, ID card copy, medical record and invoice, was kept by the receptionist and then transferred to the office of the medical assistant. All these procedures were developed as a guideline for dental service at the CHH L2 for years (Figure 2A).

After the COVID-19 epidemic in Jan, 2020, two additional procedures were added. For patients, except in an emergency, all the patients seeking for dental care need to make appointments by phone, email or text message firstly. A primary screening about the patient's chief complaint, medical history and questions directed to determine the risk of COVID-19 should be performed when scheduling an appointment. After the patient arrived at the hospital according to the appointment time, body temperature, heart rate and blood pressure were monitored firstly at the nurse station. Before entering the dental treatment room, the patients should be requested to wear a surgical mask and then screened questionnaire for COVID-19 (Figure 2B).

### Data collection

Medical records of all patients visited to the CHH L2 between October 1st 2018 and September 30th 2022 (4 rotation mission periods: 22nd, 23rd, 24th and 25th Chinese medical peacekeeping contingents) were identified, and all dental service were analyzed. The total number of patients who required dental care was based on the serial numbers. Patients visited to the CHH L2 mainly contains four cohorts: UN military, UN police, UN civilian staff, and others (including UN volunteers, International Agency, International Contractor and Officer Visitors). Dental treatments were limited to patients with emergency situation and routine oral and maxillofacial problems. Analysis of different types of dental treatment was based on soft tissue and teeth number recorded. Every single tooth problem or soft tissue problem were recorded separately. Multiple visits for treatments like root canal therapy (RCT) and following treatments of the same tooth in a single patient were also counted separately, examinations like pulp vitality test and percussion was not recorded, and only the main operation procedure was recorded. Treatments provided were classified into 6 main categories: radiographs, aesthesia, resin composite filling, RCT, extractions and other treatment (periodontics, oral surgery, rinsing, local medications and removal of stitches, etc.). Medical records of all patients were accessed and statistical analysied for the research purposes between October 1st, 2018 and December 30th, 2022.

## Results

### Overall service and visit trend analysis

**Figure 3:**
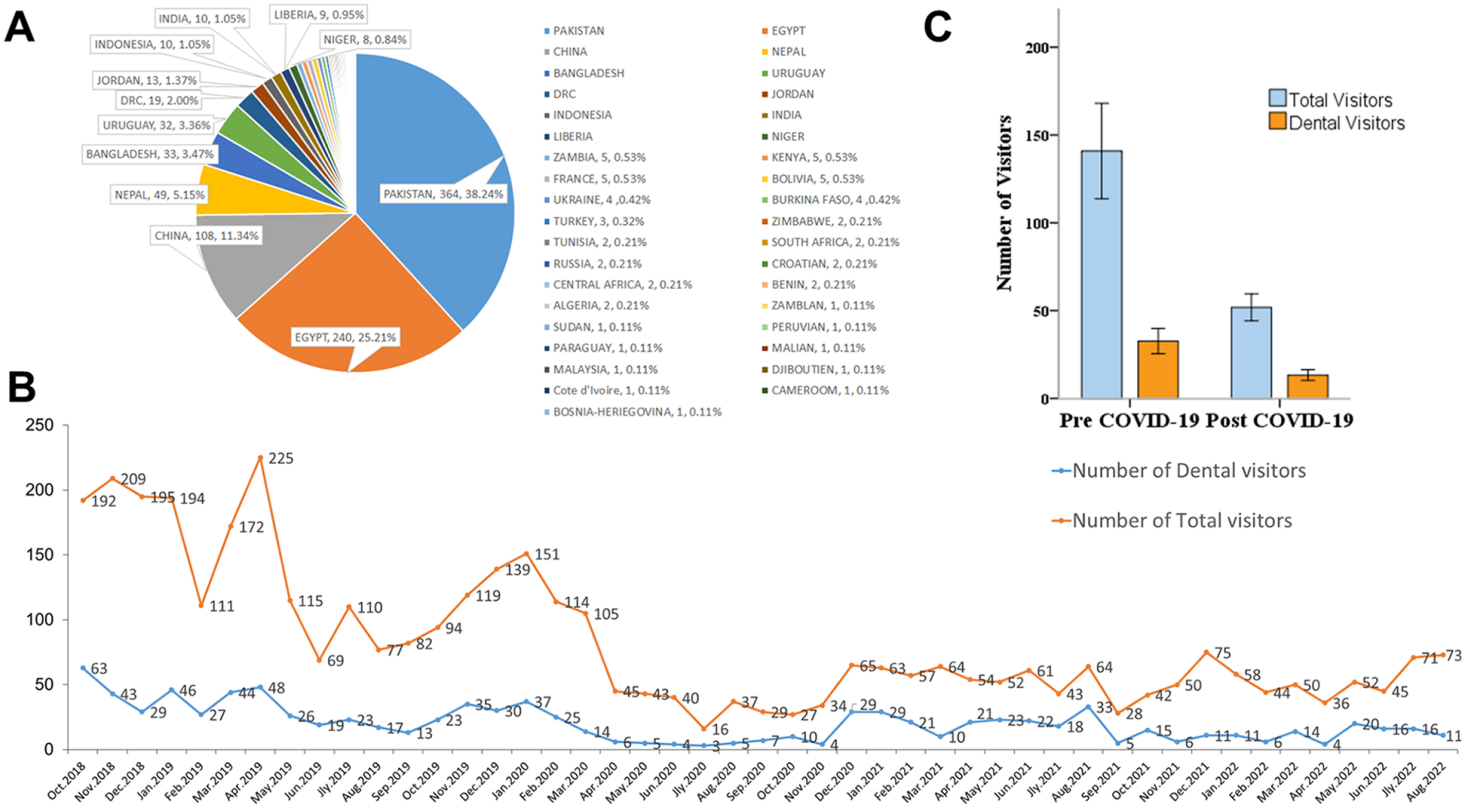
Dental visitors trend analysis from Oct. 2018 to Sep. 2022. **(A)** Dental visitors identified with nationality. **(B)** Analysis of COVID-19 on the monthly number of dental visitors. **(C)** Peak-time description of the monthly number.

During the 48-month mandate period, there were 3,913 outpatient and emergency patients in CHH L2. Dental visitors represented 952/3,913 (24.33%), including 50 females (5.25%) and 902 males (94.75%). The dental patients ranged in age from 19 to 62 years old, with an average age of 34.97 ± 7.02 years. The numbers of dental patients by identification are demonstrated in Table 1. The proportion of UN military personnel is 91.39%. These patients came from 37 countries and regions, and the top ten countries accounted for 92.23% of the dental patients, respectively: Pakistan 38.24%, Egypt 25.21%, China 11.34%, Nepal 5.15%, Bangladesh 3.47%, Uruguay 3.36%, DRC 2.00%, Jordan 1.37%, Indonesia 1.05% and India 1.05% (Figure 3A).

**Table 1:** Dental visitors to CHH L2 from Oct. 2018 to Sep. 2022.

	22nd		23rd		24th		25th		Total	
Number										
Total visitors	1,751		932		612		618		3,913	
Dental visitors	396	22.62%	198	21.24%	225	36.76%	133	21.52%	952	24.33%
Sex										
Female	15	3.79%	2	1.01%	13	5.78%	20	15.04%	50	5.25%
Male	381	96.21%	196	98.99%	212	94.22%	113	84.96%	902	94.75%
Occupation										
UN military Personnel	370	93.43%	185	93.43%	203	90.22%	112	84.21%	870	91.39%
UN police	7	1.77%	1	0.51%	2	0.89%	8	6.02%	18	1.89%
UN civilian staff	10	2.53%	11	5.56%	11	4.89%	8	6.02%	40	4.20%
UN Others	9	2.27%	1	0.51%	9	4.00%	5	3.76%	24	2.52%

The numbers of total visitors and dental visitors both showed a decreasing trend after the outbreak of COVID-19 in January, 2020, and dropped to the lowest in July 2020 and continued to November 2020 (Figure 3B). In the following 2 years, the number has been kept at a low level, which is significantly lower than that before the outbreak of COVID-19 (*p* < 0.05) (Figure 3C). One-Way ANOVA analysis shows that the number of dental visitors in each month before the outbreak of COVID-19 accounted for 23.36% ± 4.50% of all patients, which was not statistically different from the proportion after the outbreak of COVID-19 24.59% ± 10.90% (*F* = 0.354, *p* > 0.05). However, the monthly number of visits fluctuated significantly after the epidemic.

### Disease classification

Eighteen types of oral and maxillofacial diseases were taken into account, with a total of 1,116 teeth or soft tissues from 952 participants for dental care listed in Table 2. Of these, there were 1,106 cases from 870 UN military personnel; 18 cases from 18 UN police; 46 cases from 40 UN civilian staff and 27 cases from 24 other personnel. The main types of emergencies were acute toothache caused by tooth trauma, acute pulpitis, acute apical periodontitis and abscess, accounting for 13.98% (156/1,116) of the dental patients, and the rest were routine patients, no dental patients were evacuated or arranged for hospitalization. Dental caries, dentin sensitivity, pulpitis, apical periodontitis, tooth trauma, cracked tooth, residual root, residual crown and wisdom teeth problems made up 90.23% (1,007/1,116) of all oral and maxillofacial problems. Abscess, periodontitis, gingivitis and mucosa ulcers made up a small part of the care provided 8.78% (98/1,116). Restoration debonding, occlusal disturbance, injury, infection and TMJ disorders were extremely uncommon (fewer than 1%).

**Table 2:** Number of diagnoses of dental visitors from Oct. 2018 to Sep. 2022.

Disease	22nd	23rd	24th	25th	Total
Dental caries	184	34	55	48	321
Pulpitis	93	60	95	58	306
Apical periodontitis	87	33	69	18	207
Pericoronitis	19	22	17	7	65
Gum inflammation	11	22	12	1	46
Residual root	1	13	3	13	30
Residual crown	6	13	4	6	29
Periodontitis	13	7	4	2	26
Dentine sensitivity	3	10	8	2	23
Mucosa ulcers	6	4	0	3	13
Lost crown/bridge	3	3	6	1	13
Abscess	0	9	0	3	12
Fractured tooth	0	4	1	6	11
Occlusal problems	0	2	1	6	9
Cracked tooth	1	0	0	1	2
Parotid duct obstruction	0	0	0	1	1
TMJ disorders	0	0	1	0	1
Maxillofacial Infection	1	0	0	0	1

### Treatment item classification

Altogether, 2,180 dental treatments (2.29 procedures per patient) were provided during the mission period, as shown in Table 3. Treatments related to dental caries problem account for the majority of dental care. Dental radiographs taken (618, 28.30%), local anesthesia (448, 20.55%), RCT (373, 15.14%), resin composite filling (330, 15.14%) and extraction (248, 11.38%) constitute 92.48% of all the dental treatments. The remaining other 7.52% treatments mainly include abscess incision and drainage, rinsing, local medications, and restoration re-bonding, removal of stitches, etc.

**Table 3:** Number of treatments to dental visitors from Oct. 2018 to Sep. 2022.

Treatments	22nd	23rd	24th	25th	Total
Radiology	226	128	166	97	617
Anaesthesia	148	96	120	84	448
Root cannal therapy	157	62	115	39	373
Resin composite filling	166	49	64	51	330
Extraction	63	63	63	59	248
Other treatment	42	62	33	27	164

## Discussion

### Particularity of military dental service

When preparing medical service for military operations, dentistry is always inevitably need to be considered, since studies have described the occurrence of dental emergency, craniomaxillofacial injuries in military during training exercises, deployments, stabilization operations and in combat environments (17, 18). However, this medical unit faces some unique challenges to the effective delivery of healthcare to deployed troops from diverse nations, religions and cultures.

During the 48 months medical service, patients from 37 countries and regions received dental consultation and treatments, the top ten countries are related to the troops deployed in South Kivu (Figure 3A). Language misunderstanding and gender sensitivity are all potential unpleasant factors during the medical service in UN missions. Effective communication can avoid medical disputes or interpersonal conflicts, customs and habits of patients in multiple countries must be fully respected. Dentists must be more flexible, thoughtful, and rigorous when communicating about medical conditions or dental treatment, by using additional gestures, translators, or patient companions to translate, accurately convey and obtain the meaning of the patient.

Unlike civilian settings, militaries deployed to foreign grounds were always exposed to local emerging and re-emerging infectious diseases, which may spread more readily within military settings or transferred between military and communities, posing unique challenges to the prevention and control (19, 20). In addition to routine dental diagnosis and treatment, dentist should pay attention to the health status of patients and learn how to identify the oral symptoms of infectious diseases, as some infectious diseases may cause symptoms in the mouth, and the sufferers may firstly visit a dentist for help (21–23). Since fever is the most common clinical feature of many infectious diseases, including COVID-19 (present in 88.7% of the cases) (24), a noncontact forehead thermometer is greatly help for in-office patient screening. Because it is a potential cross-infection route of many infectious diseases, due to the invasive operation and droplet spattering of oral treatment. Any dental patient suffers fever or indication of suspicious infectious disease would not accept dental treatments, and would be transferred to specialists and laboratory examination.

### Prevention of COVID-19 in CHH L2

Studies have proved the generation of high amounts of droplets and aerosols during routine dental procedures was effective in spreading of COVID-19 (24). After the COVID-19 epidemic, ultrasound scaling procedures was suspended, and dental care room was relocated from the interior of the facility to outdoor to reduce the risk of cross infection. Strict protection measures against COVID-19 in visiting procedures were implemented.

For all dental patients, except in life threatening emergency, should make appointments and be screened twice to determine the risk of COVID-19, the detailed process is shown in Figure 2A. However, patients suspected of confirmed for COVID-19 may have no signs or symptoms during the incubation period while they are potential carriers and may transmit the disease to other individuals, this property makes efficient control of this disease extremely difficult (25). As infectious diseases have proven to have a significant impact on the military in the morbidity, disrupting the routines training and combat readiness, or even causing unplanned suspension of military operations in the event of an outbreak (19, 26). Therefore, daily appointments should be limited to the same department or camp as much as possible, and a separate waiting area was assigned to each group of patients to minimize the risk of potential COVID-19 transmission between different units. For some dental emergency patients without fever, and who are far away from the camp and cannot rush back that night, nucleic acid test of COVID-19 is required before staying in the ward.

In order to protect the skin and mucosa against the potential infected secretions, dentist and assistant are required to be strictly secondary protection throughout the admission and treatment process, including wearing protective hospital gown, N95 mask covered with a surgical mask, a full-face shield and double layers of surgical gloves. Besides, dentist and assistant should strictly follow the standard precautions for the contact and airborne infections and hand hygiene guidelines of two-before-and-three-after recommended by the West China Hospital of Stomatology, Sichuan University (27). After all the dental treatment, dentist and assistant are required to change protective clothing and masks in a fixed place and enter the office area through a special channel, and the operation room was effectively disinfected with epidemic prevention and disinfection personnel, including with appropriate hospital grade disinfectants such as sodium hypochlorite, UV light irradiation and facilitates ventilation. Regular water quality testing and microbiological testing were also implemented.

### Peak-times of dental service

In MONUSCO, the peacekeeping forces rotate every August and September, and the medical visits number is also cyclical. Usually, in October, November and December of each rotation period, the number of medical visits gradually increases and becomes stable, the possible reason for this was troops have adapted to the new environments and their mission and have time to receive dental care for themselves. Then there were still some cases needing dental care in August and September, but the number decreased. The maximum number of daily dental visits during the study period was 7, including 5 patients (from the same camp) by appointment and 2 in emergency, meets the requirements of the UN for dental settings in level 2 hospitals (13). Three years of COVID-19 worldwide spreading has seriously affected the medical visits of deployed troops. As shown in Figures 3B,C, all the numbers indicate monthly cases. When COVID-19 was first reported to be prevalent, the number of medical visits gradually began to decline. With the gradual deepening of the understanding of the harm of COVID-19, the number of medical visits reached the lowest 6 months after the outbreak and remained depressed for about 4 months. This trend change is closely related to the local epidemic situation. The maximum number of total visits per month is 225 (Apr. 2019) and the minimum number was 16 (Jul. 2020), while the maximum number of dental visits per month is 63 (Oct. 2018) and the minimum number was 3 (Jul. 2020). Both numbers were significantly affected by the COVID-19 epidemic. However, dentistry was the most frequently visited department during the study period, accounting for 24.33% of the total number of patients, and the ratio has not changed significantly before and after COVID-19 epidemic.

### Dental treatments in CHH L2

Dental visits for routine caries accounted for 42.99% of all dental cases in 22nd, while the ratio decreased to 14.41% (23rd), 19.93% (24th) and 27.27% (25th). Emergency cases caused by fracture, abscess, pulpitis, apical periodontitis and pericoronitis accounted for 9.81% of all dental cases in 22nd, while the ratio increased to 13.56% (23rd), 15.94% (24th) and 21.59% (25th). The ratio of routine and emergency dental visits changed between the four rotated medical teams, the reason may be managed by military camps, avoid going out in non-emergency situations, managements have been affected by the COVID-19.

To avoid serious consequences (hemorrhage, fracture) caused by dental treatment for military personnel under such simple conditions, dental radiographs were usually taken in the diagnosis of deep caries and during root canal treatments, especially before tooth extraction. Painless and comfortable is the concept of modern dental treatment, local anesthesia is carefully used when operation will cause pain to patients, that would lead to better cooperation and communication. The “four handed operations” concept was also executed throughout the missions.

### Departure recommendations

According to the limited statistical data, the number of personnel visits for dental treatment was the highest among all specialties. We advise increasing the number of dentist and diagnostic and treatment equipment such as cone beam CT, but this conflicts with the number of stuffs in UN's secondary hospitals and needs increased costs and transportation capacity. By counting the number of various diseases, showed as Table 2, the top 5 diseases were dental caries, pulpitis, periapical periodontitis, periodontitis, and gingivitis. Therefore, it is recommended to arrange dental examinations at least one month before the departure of peacekeeping personnel who are about to be dispatched. If potential problems are found, they should be treated first before dispatch. For peacekeeping personnel who have been on duty for more than a year, an oral hygiene should be arranged before departure, since the periodontal abscess situation in the disease statistics.

In addition, there are also the problem of wisdom tooth pericoronitis and residue roots, it is recommended to arrange a dental panoramic x-ray for all dispatched personnel, once there was a need for tooth extraction, you can refer to it due to equipment configuration limitations.

The disease and dental treatment programs described in this study are significant for the preparation before departure to the later rotating dentists. The only dentist should have the ability to master common professional skills of filling, endodontic interventions, periodontal treatment, extraction etc., as well as assistant training, disinfection and packaging of operating equipment, simple troubleshooting and equipment maintenance, selection and classification of dental materials etc. Only with adequate preparation can dentists be better qualified for dental service in the peacekeeping missions. After sending peacekeeping medical teams for decades, China has been at the forefront in contributing to international medical assistance and peace-keeping medical service at an international level.

## Conclusion

Military dental service is always indispensable for United Nations peacekeepers. And due to the invasive operation and droplet spattering of oral treatment, it is a potential cross-infection route of many infectious diseases, like COVID-19. So, the CHH L2 carried out a series of previewing and triage process to improve efficiency of dental care and avoid cross-infection of COVID-19 in the dental clinic simultaneously.

Dentistry was the most frequently visited department in the CHH L2, dentists must have a clear understanding of local epidemiology and scope of dental treatments, and prepare the budget contains sufficient consumables, drugs and frequently used equipment backup or maintenance parts within the validity period, to ensure the mission successfully completed.

## Data Availability

The datasets presented in this article are not readily available because the data were collected from the medical records of oral diseases of peacekeeping troops from various countries. Due to confidentiality requirements, we cannot provide personal detailed medical records. Requests to access the datasets should be directed to wangchao51115111@126.com.
